# Complex intracranial vascular complications caused by essential thrombocythemia: a critical case report

**DOI:** 10.1186/s12883-020-01986-9

**Published:** 2020-11-07

**Authors:** Jian Xie, Leiyu Geng, Baoyu Yuan, Yijing Guo, Zhijun Zhang

**Affiliations:** Department of Neurology, Affiliated ZhongDa Hospital, School of Medicine, Southeast University, Nanjing, 210009 Jiangsu China

**Keywords:** Essential thrombocythemia, Intracerebral hemorrhage, Ischemic stroke

## Abstract

**Background:**

Essential thrombocythemia (ET) is a myeloproliferative neoplasm characterized by elevated and dysfunctional platelets. ET can result in systemic thrombotic and hemorrhagic complications, and it’s a rare cause of stroke. The coexistence of multiple vascular lesions has seldom been reported in patients with essential thrombocythemia.

**Case presentation:**

A young woman presented with isolated and persistent vertigo and vomiting. The CT scan indicated a hyperdense lesion in the right cerebellar hemisphere. No signs of cerebral artery malformation were observed in the CT angiography (CTA). Besides, the blood tests indicated an increase in platelet and white blood cell counts. The patient then suddenly developed a transient unconsciousness with left horizontal nystagmus when staring to the right. The subsequent cranial magnetic resonance imaging (MRI) scans indicated a diffuse and acute infarction of the pons and hemorrhage in the bilateral cerebellums. Further digital subtraction angiography (DSA) revealed a progressive and critical intracranial vertebral arterial occlusion. The patient’s clinical condition stabilized after cytoreductive therapy with interferon-α (IFN-α), even though endovascular and antiplatelet treatments were restricted because of the simultaneous presence of intracerebral hemorrhage (ICH) and ischemic stroke. A *JAK2 V617F* mutation was later detected through genetic testing, further confirming the diagnosis of ET. The patient was treated with a continuous regimen of IFN-α, and an antiplatelet treatment (aspirin) was added after ICH. The 1-year follow-up indicated normal platelet levels and no additional stroke event.

**Conclusions:**

This case demonstrates that ET can be a rare cause of the cerebrovascular disease (CVD), even though the coexistence of ischemic and hemorrhagic complications. Underlying hematological system diseases should be taken into account when abnormal hemogram and CVD are concurrent in a patient. An early multidisciplinary diagnosis and intervention could significantly improve patient’s prognosis.

## Background

Essential thrombocythemia (ET) is an uncommon clonal hematopoietic stem cell disorder and a classic myeloproliferative neoplasm. The clinical characteristics of ET include elevated platelet count and platelet dysfunction. ET most commonly occurs in adults, with the mean age at diagnosis of 58 years. Patients with ET have a higher risk of thrombotic events, transformation to myelofibrosis or acute leukemia. Early diagnosis and treatment can significantly improve patients’ life span [1].

Systemic thrombotic and hemorrhagic lesions are common complications of ET [2]. The formations of thrombi in the cerebrovascular system can cause ischemic strokes [3], transient ischemic attacks (TIA) [4], and intracranial venous or venous sinus thrombosis [5]. Intracranial hemorrhagic complications of ET are rare and include intracerebral hemorrhage (ICH) [6] and subarachnoid hemorrhage (SAH) [7]. Early detection and treatment of ET can help prevent the recurrence of stroke and improve the resulting neurological deficits.

Here we report a case of simultaneous occurrence of ICH and ischemic stroke in a female patient due to ET, as far as we can ascertain, such a complex case with intracranial thrombotic and hemorrhagic complications in ET patient is seldom reported.

## Case presentation

**Fig. 1:**
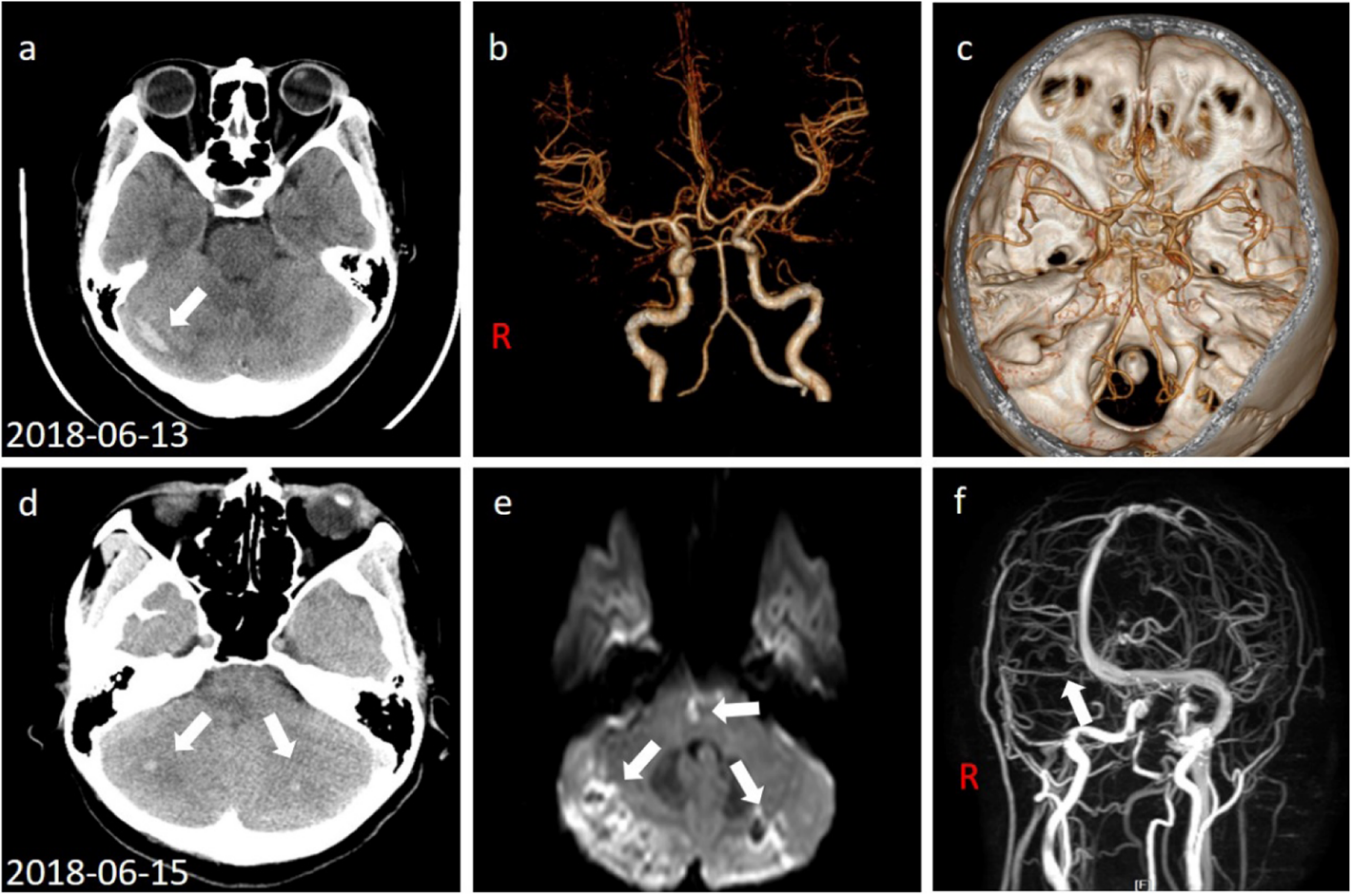
First CT and CTA scans on 2018-06-13, showed hyperdense region (white arrow) on the right cerebellum without arterial malformation (**b**, **c**). A second CT scan after the deterioration of clinical condition showed bilateral hemorrhage (white arrows) in the cerebella (**d**). Brain MRI indicated that acute lacunar infarction in pons (white arrow) with restricted diffusion on DWI (**e**). MRV indicated that the right transverse and sigmoid sinuses were thin and tortuous (**f**, white arrow)

A 41-year-old woman was admitted to the emergency department (ZhongDa hospital. Southeast University) due to a sudden onset of persistent vertigo and vomiting, which relieved while supine. She did not complain of fever, dysphagia, hearing loss, hemiparesis or hemianesthesia. The patient was a hairdresser and dyed her hair once a month for 20 years. Her history of past illness was unremarkable. The neurological examination revealed mild dysmetria on the finger-to-nose and heel-to-shin tests. No dysarthria, facial or lingual paralysis, sensory or motor abnormalities, Babinski’s or meningeal irritation sign were seen. A brain CT scans demonstrated a hyperdense lesion in the right cerebellar hemisphere. There was not cerebrovascular malformation observed on CTA (Fig. 1a ~ c). Blood routine examination showed white blood cell (WBC): 19.67 × 10^9^/L; neutrophils: 14.90 × 10^9^/L; and platelets: 729 × 10^9^/L, with normal coagulation function. The initial diagnosis was hemorrhagic stroke caused by haematological disease. Bone marrow biopsy and genetic testing were carried out the next day, after the consultation with a hematologist.

**Fig. 2:**
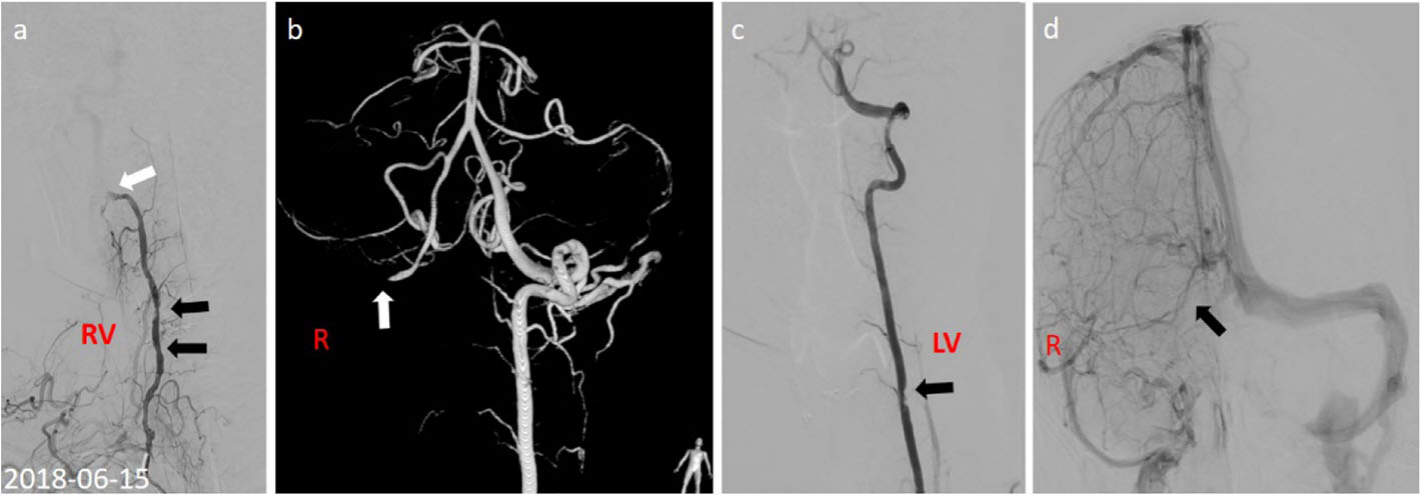
DSA displayed multiple filling defects in V1 and V2 segments of the right vertebral artery (black arrow), occlusion in V3 segment of the right vertebral artery (white arrow), and multiple filling defects (black arrow) in V1 to V2 segments of the left vertebral artery (**a**-**c**). Right transverse and sigmoid sinuses were not apparent by DSA (**d**, black arrow)

Two days later, the patient suddenly developed transient unconsciousness with cyanochroia, and her blood pressure was too low to measure, with a SpO2 of 66%, and a pulse rate of 150 beats/min. Elevation of the lower limbs, inhalation of oxygen, rehydration and vasopressor agents were administered immediately. Her clinical symptoms improved gradually 20 mins after treatment. The subsequent neurological examination revealed left horizontal nystagmus when staring to the right. The MRI scan showed diffuse and acute infarction located in the pons and bilateral hemorrhage in the cerebella. MRV indicated the stenosis and tortuosity of the right transverse and sigmoid sinuses (Fig. 1d ~ f). DSA revealed multiple filling defects in the V_1_ and V_2_ segments of bilateral artery and occlusion in V_3_ segment of the right vertebral artery, suggesting progressive critical intracranial vertebral arterial occlusion. Neither the right transverse nor the sigmoid sinuses were apparent on DSA (Fig. 2a ~ d).

**Fig. 3:**
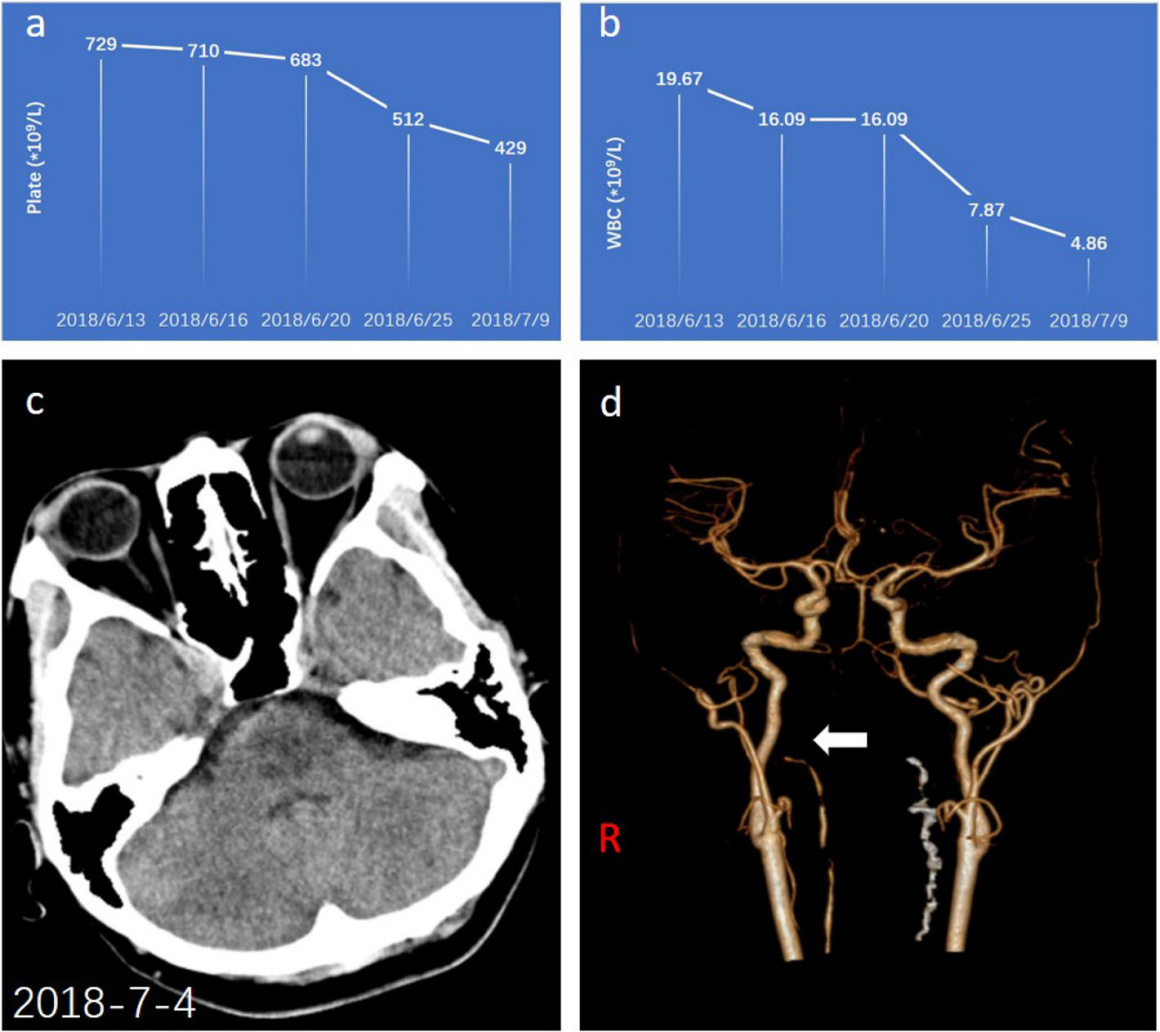
Variations of WBC and platelet counts during the whole course of treatment (**a**, **b**). CT scan after 3 weeks displayed an absence of hemorrhagic lesions (**c**), and occlusion of the right vertebral artery (**d**)

A possible culprit for the occurrence of complex cerebrovascular lesions was determined to be myeloproliferative disease after consultation with a hematologist. However, endovascular and antiplatelet treatments were not advisable due to the contradiction caused by the simultaneous presence of ICH and ischemic stroke. After treatment with IFN-α (3million U, s.c. tiw) and intensive care, the patient’s clinical condition did not deteriorate. A subsequent bone marrow smear indicated hyperplasia of the megakaryocytes. A mutation in *JAK2 V617F* and an absence of the *BCR/ABL* fusion gene were identified by genetic testing (PCR, Sanger). The diagnosis of ET was therefore confirmed according to the diagnostic criteria stated by the WHO_2016_. The patient’s condition stabilized after the treatment of INF-α with a decrease in platelet and WBC counts (Fig. 3a ~ b). The combined therapy of low-dose aspirin (100 mg, PO. daily) and INF-α (3million U, s.c. per week) was subsequently administered after the disappearance of hemorrhagic lesions (Fig. 3c ~ d) according to the high risk of thrombosis (IPSET-Thrombosis score of 4). The patient remained free of stroke and maintained normal levels of platelets throughout a 1-year follow-up period.

## Discussion and conclusions

The patient we report here presented with persistent and isolated vertigo, suggesting acute prolonged vestibular syndrome, abnormal blood count and complex cerebrovascular disease indicated rare etiology. The diagnosis of ET was further supported by her clinical manifestations, the presence of multiple intracranial vascular lesions, high platelet counts, a *JAK2 V617F* mutation and the absence of the *BCR/ABL* fusion gene [8].

ET is an infrequent cause of thrombocythemia (less than 2%) with an annual incidence of 1.2 ~ 3.0 /100000. The median age of diagnosis is 58 years (18 ~ 96), 67% of patients are female. The majority of patients with ET have *JAK2V617F, CALR* or *MPL* mutations (60, 20 and 3% respectively), and 10 ~ 20% of ET patients are triple-negative [1]. The most frequent symptoms include headache (27.5%) and abdominal or bone pain (5.5%), but half of ET patients are asymptomatic [9], and 14% of patients are diagnosed after arterial thrombosis [1]. The diagnosis of ET is established from platelet counts, bone marrow biopsy, and genetic testing, which can differentiate ET from other disorders, such as reactive thrombocytosis, chronic myelocytic leukaemia, polycythemia vera, primary myelofibrosis, myelodysplastic syndromes, or other myeloid neoplasms. The managements of patients depend on the risk stratification and IPSET-Thrombosis score, and therapeutic regimens include antiplatelet and cytoreductive therapy [10–12].

However, complications of ET can involve multiple organs leading to ischemic and hemorrhagic injuries. Ischemic stroke in patients with ET are attributed to high platelet count and the hypercoagulable state caused by higher plasm levels of P-selectin, platelet factor 4, sCD40L, decreased protein S levels and acquired protein C resistance [13, 14]. Patients with ET suffer repeated episodes of ischemic stroke or TIA without treatment, and thrombolysis with rt-PA in such patients has not been verified in a clinical trial, even though a case report has been published [14]. The combination of anticoagulant and cytoreductive therapy may be effective for ET patients with severe arterial stenosis [4, 15]. Antiplatelets and cytoreductive therapies are effective and preventive strategies for ischemic events [16]. Haemorrhagic complication in ET patients may be associated with acquired von Willebrand Syndrome (AVWS), dysfunction of platelet, and secondary venous hypertension caused by venous thrombus. High platelet counts are negatively correlated with the proteolysis of Von Willebrand Factor (VWF) multimer, which results in dysfunction of the VWF, and patients with AVWS are prone to experience bleeding complications if not treated. Measurements of VWF:Ag, FVIII, and ristocetin cofactor activity (RCoA) levels contribute to the diagnosis of AVWS [17, 18]. Dysfunction of platelet also results in bleeding [12]. Moreover, leukocytosis plays a dual effect on both thrombosis and bleeding events, and an increase and activation of neutrophils have been associated with impairment of the blood-brain barrier and the extracellular matrix [18–21]. ICH is rare in patients with ET, and expectant management or surgical treatment in such cases depends on the severity of the disease. However, cytoreductive therapy is not contraindicated [6, 22]. SAH also occurs in patients with ET, DSA should be routinely performed in patients suspected to have intracranial aneurysms, and the treatment of an aneurysm is the primary aim [7, 23]. There are some reports of cerebral venous or venous sinus thrombosis in patients with ET. The presence of a *JAK2 V617F* mutation is linked to such thrombosis, and antithrombotic therapy has been shown to be effective in some patients [5, 24].

In this report, some strengths should be addressed. Firstly, the clinical manifestations, the neuroimaging results, increased WBC and platelet counts, and a mutation in *JAK2 V617F* and a negative *BCR/ABL* fusion gene confirmed the diagnosis of stroke caused by ET. Secondly, the coexistence of recurrent ICH and ischemic stroke is seldom reported in an ET patient, and the presence of ICH is believed to be closely related to an increase in neutrophil granulocyte, dysfunction of platelet, and possible AVWS. However, we did not check the serum level of VWF to confirm the AVWS. Thirdly, the presence of multiple thrombi inside the vertebral arteries rendered the situation complex and critical, and thrombolysis or interventional therapies were contraindicated due to hemorrhage. Early treatment with IFN-α was effective in mitigating the patient’s condition. Finally, the occupation and living habits of the patient led to a long term exposure to toxic and harmful chemicals, which could constitute a risk factor for ET [25, 26]. There are some limitations to our treatment. We did not check more markers of thrombo-hemorrhagic homeostasis, such as plasma levels of VWF, RCoA, and P-selectin. Therefore the mechanisms of ICH and infarction could not be definite. Furthermore, the cause of the patient’s shock cannot be attributed to brain lesions. We deduced that it might be the result of possible transient ischemia of the cardiovascular center in brainstem.

In conclusion, our case highlights that the coexistence of elevated platelet counts and cerebrovascular disease is suggestive of probable ET. Genetic testing should also be performed to confirm suspected ET. Early intervention could prevent an unfavorable prognosis. Moreover, platelet counts should be monitored periodically.

## Data Availability

Not applicable.

## Abbreviations

ET: Essential thrombocythemia
CTA: Computed tomography angiography
MRI: Magnetic resonance images
DSA: Digital subtraction angiography
ICH: Intracerebral hemorrhage
IFN-α: Interferon-α
CVD: Cerebrovascular disease
TIA: Transient ischemic attack
MRV: Magnetic resonance venography
IPSET: International Prognostic Score for Essential Thrombocythemia
AVWS: Acquired von Willebrand Syndrome
VWF: Von Willebrand Factor
